# Supplementary material for: Structure and dynamics of endogenous cardiac troponin complex in human heart tissue captured by native nanoproteomics
Source: Nat Commun. 2023 Dec 18;14:8400. doi: 10.1038/s41467-023-43321-z (PMC10728164; doi:10.1038/s41467-023-43321-z)
Supplement: Supplementary file 3 — Description of Additional Supplementary Files [file 41467_2023_43321_MOESM3_ESM.pdf]

Description of Additional Supplementary Files

File Name: Supplementary Data 1
Description: Summary of cardiac troponin (cTn) proteoforms identified during the nTDMS analysis.
