# Supplementary material for: Structure and dynamics of endogenous cardiac troponin complex in human heart tissue captured by native nanoproteomics
Source: Nat Commun. 2023 Dec 18;14:8400. doi: 10.1038/s41467-023-43321-z (PMC10728164; doi:10.1038/s41467-023-43321-z)
Supplement: Supplementary file 5 — Reporting Summary [file 41467_2023_43321_MOESM5_ESM.pdf]

nature portfolio

Corresponding author(s): Ying Ge and Song Jin

Last updated by author(s): Oct 24, 2023

Reporting Summary

Nature Portfolio wishes to improve the reproducibility of the work that we publish. This form provides structure for consistency and transparency in reporting. For further information on Nature Portfolio policies, see our Editorial Policies and the Editorial Policy Checklist.

Statistics

For all statistical analyses, confirm that the following items are present in the figure legend, table legend, main text, or Methods section.

n/a	Confirmed	
☐	☒	The exact sample size (*n*) for each experimental group/condition, given as a discrete number and unit of measurement
☐	☒	A statement on whether measurements were taken from distinct samples or whether the same sample was measured repeatedly
☐	☒	The statistical test(s) used AND whether they are one- or two-sided<br>*Only common tests should be described solely by name; describe more complex techniques in the Methods section.*
☒	☐	A description of all covariates tested
☒	☐	A description of any assumptions or corrections, such as tests of normality and adjustment for multiple comparisons
☐	☒	A full description of the statistical parameters including central tendency (e.g. means) or other basic estimates (e.g. regression coefficient) AND variation (e.g. standard deviation) or associated estimates of uncertainty (e.g. confidence intervals)
☐	☒	For null hypothesis testing, the test statistic (e.g. *F*, *t*, *r*) with confidence intervals, effect sizes, degrees of freedom and *P* value noted<br>*Give P values as exact values whenever suitable.*
☒	☐	For Bayesian analysis, information on the choice of priors and Markov chain Monte Carlo settings
☒	☐	For hierarchical and complex designs, identification of the appropriate level for tests and full reporting of outcomes
☒	☐	Estimates of effect sizes (e.g. Cohen's *d*, Pearson's *r*), indicating how they were calculated

Our web collection on statistics for biologists contains articles on many of the points above.

Software and code

Policy information about availability of computer code

Data collection	Mass spectrometry data was collected using otofControl v. 4.3 and ftmsControl v. 2.1.0.
Data analysis	Mass spectrometry data was analyzed using Compass DataAnalysis v. 4.3 and DataAnalysis v. 5.1. Mash Native v. 1.1. was used for fragment ion validation. Plots were generated in Microsoft Excel 2023 and figures were made in Microsoft Powerpoint 2023 or Adobe Illustrator CC 2023.

For manuscripts utilizing custom algorithms or software that are central to the research but not yet described in published literature, software must be made available to editors and reviewers. We strongly encourage code deposition in a community repository (e.g. GitHub). See the Nature Portfolio guidelines for submitting code & software for further information.

Data

Policy information about availability of data

All manuscripts must include a data availability statement. This statement should provide the following information, where applicable:

- Accession codes, unique identifiers, or web links for publicly available datasets
- A description of any restrictions on data availability
- For clinical datasets or third party data, please ensure that the statement adheres to our policy

The mass spectrometry proteomics data generated in this study have been deposited to the ProteomeXchange Consortium via the PRIDE partner repository under the accession code PXD042825 and MassIVE repository with under the accession code MSV000092130 (ftp://MSV000092130@massive.ucsd.edu/). In addition, all the raw data files or spectra are available upon request. The structures corresponding to the PBD-codes mentioned in the main text are available through these

links: Figures 1 and 4: 1J1E (https://doi.org/10.2210/pdb1J1E/pdb). Source data are provided with this paper.

Research involving human participants, their data, or biological material

Policy information about studies with human participants or human data. See also policy information about sex, gender (identity/presentation), and sexual orientation and race, ethnicity and racism.

Reporting on sex and gender	Volunteers are of mixed gender (2M, 3F) (Supplementary Table 1). No gender-based analyses were performed due to our study not being designed to analyze sex/gender-based differences. Instead, our study is a development of a particular platform in which we apply to a small sample size as proof-of-concept.
Reporting on race, ethnicity, or other socially relevant groupings	Our study does not report on race, ethnicity, or other socially relevant groupings. Race, ethnicity, or other socially relevant groupings were not included in the de-identified clinical data.
Population characteristics	Volunteers are of mixed gender (2M, 3F) and of variable age (43-65 years).
Recruitment	The donor hearts were collected by the University of Wisconsin Organ and Tissue Donation-Surgical Recovery and Preservation Services. All human heart tissues were voluntarily obtained from brain-dead donors with no history of heart diseases but unsuitable for heart transplant. All samples were completely de-identified before analysis. Thus we anticipate minimal self-selection bias.
Ethics oversight	The procedures for the collection of human donor heart tissues were reviewed and approved by the University of Wisconsin - Madison Institutional Review Board (Protocol number 2013-1264).

Note that full information on the approval of the study protocol must also be provided in the manuscript.

Field-specific reporting

Please select the one below that is the best fit for your research. If you are not sure, read the appropriate sections before making your selection.

- [x] Life sciences
- [ ] Behavioural & social sciences
- [ ] Ecological, evolutionary & environmental sciences

For a reference copy of the document with all sections, see nature.com/documents/nr-reporting-summary-flat.pdf

Life sciences study design

All studies must disclose on these points even when the disclosure is negative.

Sample size	Sample sizes were not predetermined by statistical methods due to the nature of this study. To establish NP-Pep native enrichment reproducibility, we used a sample size of n=3 inter-batch syntheses and performed experiments (Fig. S2) using SDS-PAGE and RPLC-MS (Figure S5, n=3 independent tissue extractions/enrichments) to confirm reproducibility. Error bars represent standard error of the mean. Groups were considered significantly different by paired-students t-tests with $p < 0.01$. For SEC-OBE native MS experiments, we used n = 3 technical replicates to confirm reproducibility of our method (Fig. S7). For all other MS data, experiments were conducted with an average of 10-100's scans (technical repeats). At least n = 2 biological replicates were considered sufficient to demonstrate the new technical capability, which is the main purpose of our native nanoproteomics method.
Data exclusions	For all top-down MS data analysis, the following parameters were set in DataAnalysis: quality factor: 0.4, S/N: 3. Data that did not meet these requirements were excluded to ensure that only quality proteoform spectra were used data reporting.
Replication	In all of the experiments at least 3 technical replicates were used and data usually show one experiment that is representative of at least 2 independent experiments. The precise MS parameters are defined for all experimental data in the Methods section of the manuscript. All the attempts of replication were successful.
Randomization	Randomization of the order of samples for RPLC-MS analysis was performed to minimize batch effects. All attempts of technical replicates were successful. Randomization was not applicable/appropriate for all other experiments due to sample sizes not being large enough.
Blinding	Blinding was not applicable to/or appropriate for this study, since any prior knowledge does not have an effect on the validity of the outcome in this study.

Reporting for specific materials, systems and methods

We require information from authors about some types of materials, experimental systems and methods used in many studies. Here, indicate whether each material, system or method listed is relevant to your study. If you are not sure if a list item applies to your research, read the appropriate section before selecting a response.

Materials & experimental systems

n/a	Involved in the study
☒	☐ Antibodies
☒	☐ Eukaryotic cell lines
☒	☐ Palaeontology and archaeology
☒	☐ Animals and other organisms
☒	☐ Clinical data
☒	☐ Dual use research of concern
☒	☐ Plants

Methods

n/a	Involved in the study
☒	☐ ChIP-seq
☒	☐ Flow cytometry
☒	☐ MRI-based neuroimaging
